# Prevalence of *BRCA1* and *BRCA2* pathogenic sequence variants in ovarian cancer patients in the Gulf region: the PREDICT study

**DOI:** 10.1186/s12885-021-09094-8

**Published:** 2021-12-20

**Authors:** Fathi Azribi, Ehab Abdou, Emad Dawoud, Mohamed Ashour, Amgad Kamal, Mohamed Al Sayed, Ikram Burney

**Affiliations:** 1 Tawam Hospital, Al Ain, United Arab Emirates; 2 Kuwait Cancer Control Center, Kuwait City, Kuwait; 3 Medical Affairs Department, AstraZeneca, Dubai, United Arab Emirates; 4 Sultan Qaboos University Hospital, Seeb, Oman

**Keywords:** Gynaecological oncology, Cancer genetics, Epidemiology

## Abstract

**Background:**

Patients with pathogenic sequence variants (PSVs) in *BRCA1/BRCA2* are at high risk of developing ovarian cancer (OC). However, genetic testing for *BRCA1/BRCA2* PSVs is still not a routine practice in the Middle East. With the lack of epidemiological studies in the region, we aim to describe the prevalence of *BRCA1/BRCA2* PSVs in patients with OC across different countries in the Gulf region.

**Methods:**

The PREDICT study was an observational, prospective, epidemiological study, which consecutively recruited women with ovarian, primary peritoneal, and fallopian tube cancers from the following Gulf countries over the period from July 2017 to July 2019; United Arab Emirates (UAE), Kuwait, and Oman. The study was approved by the local ethics committee of participating centers. The *BRCA1/BRCA2* PSVs were assessed by tissue genetic testing using next-generation sequencing (NGS).

**Results:**

A total of 105 women were included with a median age at diagnosis of 52 years (IQR 44.5 – 61.0). Nearly 11.4% of patients reported a family history of ovarian or breast cancer, while 4.7% of patients reported a family history of other cancers. Most of the patients (70.3%) had advanced disease (FIGO stage III/IV) at presentation. Eighty-eight patients (84%) were successfully tested for somatic *BRCA1/BRCA2* PSVs. Fifteen patients (17%) were found to have PSVs in either *BRCA1*, *BRCA2*, or both genes; of them, 10 patients (11.2%) had *BRCA1* somatic PSVs alone, eight patients (9.1%) had *BRCA2* somatic PSVs, while three patients (2.9%) had both PSVs. Five patients with *BRCA1/BRCA2* somatic PSVs had germline PSVs tests, and three of them tested positive. Concerning treatment, 87.6% of patients received perioperative chemotherapy and 6.6% as first-line palliative chemotherapy. Eighty-seven (82.9%) patients underwent debulking surgery, with no residual disease in 42.5% of patients.

**Conclusion:**

Our study showed that the prevalence of *BRCA1/BRCA2* somatic PSVs in patients with OC is higher than the reported global figures (2-8%). However, more studies are warranted to further elucidate the prevalence of *BRCA1/BRCA2* somatic and germline PSVs, as well as other relevant genetic alterations, to better understand their impact on OC patient outcomes in Gulf countries.

**Trial registration:**

NCT03082976.

**Supplementary Information:**

The online version contains supplementary material available at 10.1186/s12885-021-09094-8.

## Introduction

Ovarian cancer is considered to be the eighth most common cancer among females and the eighth cause of death from cancer in women [1, 2], with a high mortality rate (62.5%) and one of the worst prognoses among gynecologic cancers [1, 3, 4]. Approximately 75% of women at diagnosis presented with advanced disease status [5], which account for the poor prognosis of the disease [5, 6]. In the Middle East, women with gynecological cancers tend to seek medical advice very late in the course of their disease due to several factors, including stigma and the lack of awareness about reproductive health [7]. Several risk factors are incorporated in the development of ovarian cancer, such as family history, indicating the involvement of a genetic component in the development of this disease [8]. Accordingly, knowledge of the genetic susceptibility to ovarian cancer can be useful in prevention efforts [9].

An important factor that contributes to ovarian cancer susceptibility is mainly represented in *BRCA1* and *BRCA2* genes that are associated with different risks of developing breast and ovarian cancers [9, 10]. Carriers of inherited pathogenic sequence variants (PSVs) in *BRCA1* and *BRCA2* are at an increased lifetime risk of developing ovarian cancer; recent reports highlighted that the cumulative risk of ovarian cancer to age 80 years was 44% for *BRCA1* carriers and 17% (95% CI, 11-25%) for *BRCA2* carriers [11]. The prevalence of *BRCA1* and *BRCA2* PSVs vary worldwide depending on several factors, including the type of cancer and ethnic groups. Germline *BRCA1* and *BRCA2* PSVs may be prevalent in up to 20% of epithelial ovarian cancer. Somatic PSVs occur less frequently in *BRCA1* and *BRCA2* PSVs, with an estimated prevalence of 2 and 8%, respectively [8, 12–14]. .There is a scarcity of data in the Middle East about the proportion of ovarian cancer patients carrying these PSVs, and genetic testing is often not part of routine practice, despite its potential for personalized medical treatment [15–18].

This study estimated the prevalence of *BRCA1* and *BRCA2* PSVs among female patients ≥18 years of age that are diagnosed with ovarian cancer, peritoneal cancer, and fallopian tube cancer in the Gulf region, with the aim of informing clinical practice and improving clinical treatments in the future. It also describes the demographics and clinical characteristics of patients with ovarian cancer.

## Methods

We followed STROBE guidelines for cross-sectional study during the preparation of the present manuscript. The study was approved by the local ethics committee of participating centers.

### Study design

This study is a multicenter, prospective, observational epidemiologic study conducted across the Gulf region in UAE, Oman, and Kuwait. Patients were recruited consecutively from Kuwait Cancer Control Center (KCCC) in Kuwait, SQUH in Oman, and Tawam Hospital in UAE over the period from July 2017 to July 2019. Female patients aged 18 years or older with histologically confirmed epithelial ovarian, primary peritoneal, or fallopian tube cancers were eligible for the study. An availability of paraffin-embedded archived tumor tissue block or a minimum of twenty 10-μm sections (if a block is not available) was also required. Patients with previous tumors metastasizing to the ovary, patients with secondary malignancies associated with ovarian cancer, patients diagnosed with other severe acute or chronic medical or psychiatric conditions were excluded from this study. Patients enrolled in this study signed an informed consent form (ICF) when meeting all inclusion criteria. Demographic and clinical characteristics data were collected from patient medical records of the hosting institutions. Archived tumor samples were also obtained for the analysis of somatic *BRCA* PSVs. Blood samples for genetic testing of germline *BRCA* PSVs were sent to a central lab.

The analysis was performed on genomic DNA isolated from formalin-fixed paraffin-embedded (FFPE) tissue from blocks or slides of the ovarian, fallopian tube, or peritoneal serous carcinoma using the Tumor *BRCA* Analysis test. As a quality check, the portion of the tumor should have measured at least 5 × 5 mm and contain at least 20% tumor cellularity. In cases where blocks were not available, one 4-5 μm H&E slide and four consecutive 10 μm unstained slides were acceptable. Patient DNA was extracted and purified from the tumor specimen, assigned a unique bar-code for robotic-assisted continuous sample tracking, and submitted for molecular testing.

The test consists of sequencing and large rearrangement analyses of the *BRCA1* and *BRCA2* genes using next-generation sequencing (NGS). For *BRCA1*, full sequence determination of approximately 5.400 base-pairs comprising 22 coding exons and approximately 750 introns were performed, excluding exons 1 and 4, which are non-coding. For *BRCA2*, full sequence determination of approximately 10.200 base-pairs comprising 26 coding exons and approximately 900 introns were performed, excluding exon 1, which is non-coding. Genomic DNA derived from the tumor was also analyzed for large rearrangement analyses by NGS to determine copy number abnormalities indicative of deletion or duplication PSVs. Somatic *BRCA* PSV was analyzed using BRCAnalysis® (Myriad Genetics, Inc., Salt Lake City, UT) kits on available tissue blocks from the subjects enrolled. Germline *BRCA* PSV using blood sample was used to detect if hereditary reason exists in those who were mutated.

Study Subjects were classified according to treatment status at the time of enrolment into treatment-experienced and treatment naïve subgroups.

### Statistical methods and data quality

Prevalence of *BRCA* PSVs and associated 95% confidence intervals were estimated from the results of genetic tests. Appropriate descriptive statistics were used to summarize the demographics and clinical characteristics of the patients as well as disease management patterns. Statistical analysis was performed using IBM SPSS. It was determined that the sample size of 120 would be sufficient to achieve a width of 15%, provided the prevalence of somatic *BRCA* PSVs is within the expected range of 15-20% [18]. PASS software was used to calculate the sample size required for our study. The potential impact of non-random assignment was reduced by attempting to recruit participants consecutively and by using consistent inclusion/exclusion criteria across all study sites.

## Results

### Demographic, clinical, and histological characteristics

In total, 105 consenting and eligible patients were enrolled over 18 months. The median age at diagnosis was 52 years (IQR 44.5 – 61.0). Approximately 68% of patients were of Arab origin. High-grade serous carcinoma was the most reported histological type of tumors (46.7%), followed by unclassified serous carcinoma (23.8%) and low-grade serous carcinoma (2.9%). More than half of the patients (54.3%) had advanced disease (FIGO stage III/IV) at presentation. Besides, 87 (82.9%) patients underwent debulking surgery, with no residual disease in 42.5%, optimal cytoreduction in 29.9%, and suboptimal cytoreduction in 25.3% of patients. The demographic, clinical, and histological characteristics of the patients are listed in Table 1.

**Table 1:** Patients Demographics

Patients’ Demographics	N	%
**Age** (Mean, SD in years)	55.2 (12.0)	
**BMI** (Mean, SD in kg/m2)	28.9 (5.7)	
**Smoking**		
Current or past smoker	3	2.9
Never smoker	102	97.1
**Race**		
Arabic	72	68.6
South Asian	22	21.0
East Asian	7	6.7
Caucasian	1	1.0
African	3	2.9
**Nationality**		
Egyptian	18	17.1
Kuwaiti	18	17.1
Omani	13	12.4
Emiratis	12	11.4
Pakistani	11	10.5
Filipinos	8	7.6
Indian	6	5.7
Others^a^	19	9.5
**FIGO classification**		
IA	4	3.8
IB	0	0
IC	14	13.3
IIA	6	5.7
IIB	4	3.8
IIC	1	1.0
IIIA	4	3.8
IIIB	5	4.8
IIIC	37	35.2
IV	11	10.5
Unknown/missing	10/9	18.1
**Histological Type**		
High grade serous carcinoma	49	46.7%
Low grade serous carcinoma	3	2.9%
Serous carcinoma (Not classified)	25	23.8%
Endometrioid carcinoma	8	7.6%
Adenosarcoma	8	7.6%
Mixed carcinoma	4	3.8%
Clear cell carcinoma	2	1.9%
Carcinosarcoma	1	1.0%
Mucinous carcinoma	1	1.0%
Müllerian mixed tumor	1	1.0%
Serous borderline tumor, micropapillary variant	1	1.0%
Missing	2	1.9%

^a^Others were 3 from Syria, 2 from each of Bangladesh, Lebanon, Sudan, and Yemen, and 1 from each of United Kingdom, Afghanistan, Comoro islands, Iran, Jordan, Palestine, Romania, and unknown origin

### Family history

A total of 10 (9.5%) patients reported having a total of 12 family members with a history of ovarian or breast cancer. Another three (2.9%) patients reported having a total of five members with other types of cancer. Those patients with family history of ovarian or breast cancer were younger than those who did not report any family history of ovarian or breast cancer (48.9 versus 55.8 years old respectively). As compared with patients who didn’t report any family history of ovarian or breast cancer, those who did were diagnosed at a younger age (46.3 vs 53.3 years). The distribution of the family history of cancers is present in Additional Table 1.

### Prevalence of *BRCA* PSVs

**Fig. 1:**
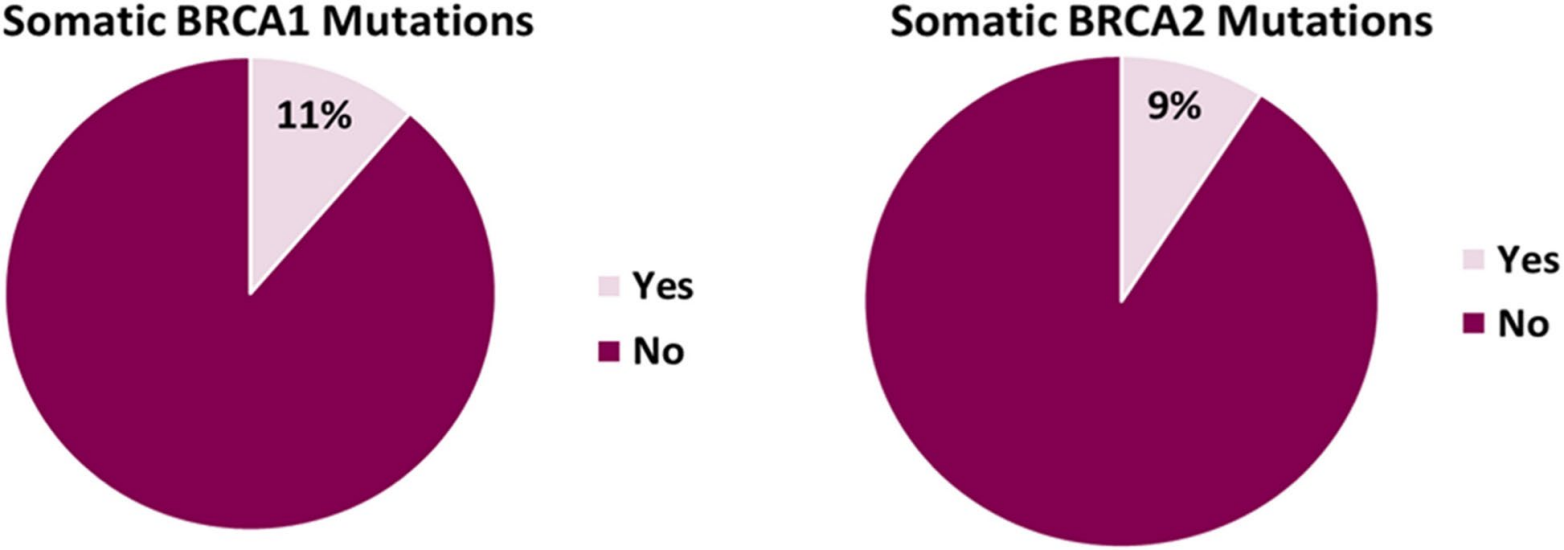
Proportion of patients with *BRCA1, BRCA2* PSVs

Somatic *BRCA* PSVs were present in 15 out of 88 subjects who had available test results (17.0, 95% CI, 9.9 to 26.6%). The individual prevalence of *BRCA1* and *BRCA2* somatic PSVs among those with test results was 11.2% (95% CI, 4.7 to 17.8%) and 9.1% (95% CI, 3.1 to 15.1%), respectively (Fig. 1). Among all 15 patients with the somatic PSVs, five patients were tested for germline PSVs. Among this group, 3 (60%) had positive germline PSVs. The list of PSVs position as per Gene Report is shown in Table 2.

**Table 2:** List of PSVs Position as Per Gene Report

- The clinically significant variant NM_007294: c.1016delA was identified in exon 10 of the BRCA1 gene. No Pathogenic BRCA2 mutation or rearrangment detected	
- Pathogenic mutation in BRCA1 gene. c.140G > T (p.Cys47Phe)	
- c.68_69del (p.Glu23Valfs*17)	
- c.3436_3439 delTGTT P.CYS1146FS	
- c.2269del (p.Val757Phefs*8) in BRCA 1	
- BRCA1: c.4096G > C(p.Gly1366Arg), variant of uncertain significance BRCA2: c.10150C > T(p.Arg3384Ter), variant of uncertain significance	
- BRCA1: c.2521C > T p.Arg841Trp BRCA1: c.1723G > A p.Glu575Lys	
- BRCA1: c.237del (p.Phe79Leufs*9) BRCA2: None Detected	
- BRCA1 mutation c.213-1A > T	
- BRCA1 c.5107-5108delGAinsTG p.Glu1703Ter likely pathogenic	
- c.4065_4065del	
- BRCA1: pathogenic mutation detected. c.1716delA, p. (Glu572*) BRCA2: None Detected	
- BRCA1 gene deleterious mutation c.4658del (p.Leu1553Cysfs*6)	
- BRCA1 gene deleterious mutation c.1140dupG(p.Lys381 Glufs*3)	
- C.512dupT (p.Gln172Thrfs*10)	
- c.441 + 1G > A	
- c.4065_4068del (p.Asn1355Lysfs*10)	
- c.367C > T(p.Arg1203)	
- c.3339 T > G, p.Tyr1113Ter pathogenic	
- No Pathogenic BRCA1 or BRCA2 Mutation detected in tumor. Additional findings of BRCA2: c.353G > T (p.Arg118Leu) variant of uncertain significance	
- c.6385G > T(pathogenic) p.Glu2129Ter	
- Positive for a deleterious BRCA2 mutation in tumor c.7618-2A > G	
- BRCA2 c.6815G > A p. (Arg2272Lys), variant may or may not modify protein function	
- BRCA2 C.3201 del (p.Val1068 Tyrfs*9) gBRCA2 c.5290_5291delTC (p.Ser1764Lysfs*3) c.9019 > T(p.Arg3007*)	

The clinical and histological features for those with somatic BRCA1/BRCA2 compared with those who do not are presented in Table 3. Patients with somatic PSVs had slightly higher prevalence of family history of cancer (13.3% versus 9.7%) and high-grade serous carcinoma (66.7% versus 38.7%).

**Table 3:** Clinical and Histological Characteristics for those with and without somatic BRCA1 or BRCA2 PSVs

	Somatic BRCA1 or BCRA2			
	No (***N*** = 72)		Yes (***N*** = 15)	
	n	%	n	%
**Age at diagnosis**				
Mean ± SD	51.90	12.10	55.73	9.95
Median (Q1-Q3)	51.50	44.50-60.0	56.00	47.0-64.0
**Family History**				
Yes	7	9.7%	2	13.3%
No	65	90.3%	13	86.7%
**Laterality**				
Bilateral	56	77.8%	10	66.7%
Unilateral	16	22.2%	5	33.3%
**Allele**				
Not Wild	17	23.6%	12	80.0%
Wild	55	76.4%	3	20.0%
**Stage at Diagnosis**				
Unknown	4	5.6%	0	0.0%
0	1	1.4%	1	6.7%
I	13	18.1%	3	20.0%
IIA	4	5.6%	2	13.3%
IIB	4	5.6%	1	6.7%
IIIA	6	8.3%	1	6.7%
IIIC	26	36.1%	5	33.3%
IV	14	19.4%	2	13.3%
**Tumor stage**				
Unknown	4	5.6%	0	0.0%
Tis	1	1.4%	0	0.0%
T1a	4	5.6%	0	0.0%
T1b	1	1.4%	0	0.0%
T1c	9	12.5%	3	20.0%
T2a	4	5.6%	1	6.7%
T2b	5	6.9%	0	0.0%
T2c	1	1.4%	1	6.7%
T3a	11	15.3%	4	26.7%
T3b	3	4.2%	1	6.7%
T3c	24	33.3%	4	26.7%
Tx	5	6.9%	1	6.7%
**Node stage**				
Unknown	4	5.6%	0	0.0%
N0	17	23.6%	2	13.3%
N1	8	11.1%	4	26.7%
Nx	43	59.7%	9	60.0%
**Metastatic stage**				
Unknown	4	5.6%	0	0.0%
M0	25	34.7%	5	33.3%
M1	6	8.3%	2	13.3%
Mx	37	51.4%	8	53.3%
**FIGO classification**				
IA	3	4.2%	0	0.0%
IB	0	0.0%	0	0.0%
IC	11	15.3%	3	20.0%
IIA	4	5.6%	2	13.3%
IIB	3	4.2%	1	6.7%
IIC	1	1.4%	0	0.0%
IIIA	3	4.2%	0	0.0%
IIIB	3	4.2%	1	6.7%
IIIC	24	33.3%	4	26.7%
IV	5	6.9%	3	20.0%
Unknown	15	20.8%	1	6.7%
**ECOG Performance status**				
0	47	65.3%	9	60.0%
1	17	23.6%	5	33.3%
2	5	6.9%	1	6.7%
3	3	4.2%	0	0.0%
**Tumor type**				
High grade serous carcinoma	29	38.7%	10	66.7%
Low grade serous carcinoma	3	4.0%	0	0.0%
Serous carcinoma (Not classified)	20	26.7%	2	13.3%
Endometrioid carcinoma	5	6.7%	1	6.7%
Adenosarcoma	5	6.7%	0	0.0%
Mixed carcinoma	4	5.3%	0	0.0%
Clear cell carcinoma	2	2.7%	0	0.0%
Carcinosarcoma	0	0.0%	1	6.7%
Mucinous carcinoma	1	1.3%	0	0.0%
Müllerian mixed tumor	1	1.3%	0	0.0%
Serous borderline tumor, micropapillary variant	1	1.3%	0	0.0%
Unclassified	1	1.3%	1	6.7%

## Discussion

The overall prevalence of somatic *BRCA1/BRCA2* PSVs in our study was 17% (95% CI, 9.9 to 26.6%). According to previously published literature, the prevalence of *BRCA1/BRCA2* PSVs ranges from 16 to 40% [13, 19–21], which reflects that the prevalence of *BRCA1/BRCA2* PSVs in the Gulf region falls within the lower end in the global prevalence range. Several studies reported variation in the worldwide prevalence of *BRCA1* and *BRCA2* PSVs [13, 19–21]. This could be due to the ethnic differences in the studied populations, barriers to access, different tissue histology, and other latent variables. The Integrated genomic analyses of ovarian carcinoma study conducted through the Cancer Genome Atlas (TCGA) Research Network reported a prevalence of 20% *BRCA* PSVs in high-grade serous ovarian cancer samples. Somatic *BRCA* PSVs were found in only 3% of patients [19]. Other studies reported an even higher overall prevalence of *BRCA* PSVs in ovarian cancer patients; In a study of 100 samples of ovarian carcinoma, the PSVs were identified in 28% of samples [22]. However, other studies found a lower prevalence of *BRCA1/BRCA2* PSVs, ranging from 8 to 14.1% [10, 23, 24]. Moreover, Koczkowska et al. reported the overall prevalence of somatic *BRCA1/BRCA2* PSVs was 4.1%, respectively [22].

Looking closely at individual *BRCA* PSVs, our study reported that *BRCA1* and *BRCA2* PSVs were detected in 11.2 and 9.1% of the patients, respectively. Variability in the prevalence of individual *BRCA1* and *BRCA2* PSVs (similar to the overall prevalence of *BRCA1/BRCA2* PSVs) was also observed in similar studies. Previous reports showed that the prevalence of somatic *BRCA1* and *BRCA2* PSVs occurred in 2 and 8% of the patients, respectively [8, 12].

Most patients in our study were with advanced stages at diagnosis. This is consistent with the study by Heintz et al., which reported that 75% of patients with ovarian cancer were typically diagnosed at late stages (56.3% stage III or higher; 44.6% stage III and 11.7% stage IV) [25]. Another German study conducted on 1038 patients with ovarian cancer showed similar results (79% presenting with FIGO III disease) [26]. The late presentation of ovarian cancer in the Gulf region and the Middle East is due to the stealthy nature of ovarian cancer, the stigma of reporting gynecological symptoms, and lack of health awareness, especially among older women.

According to Stratton et al., and compared to women without familial history of ovarian cancer, women with a family history are reported to have a higher risk of developing this disease [27]. This has been interpreted as a hereditary susceptibility genetically transmitted across generations [28]. A study by Negri et al. confirms that a family history of ovarian cancer in first-degree relatives increases the risk of ovarian cancer. A family history of a few other cancer sites, including breast, intestine, stomach, and lymphomas, was also directly associated with ovarian cancer risk, and the OR was increased for family history of any cancer [29]. A study in Saudi Arabia reported that 20.3% of cases had a positive family history of either breast or ovarian cancer in either first- or second-degree relatives. 32.5% of cases were early-onset (age at diagnosis < 40 years) [30].

A recent study by Siraj et al. in 2019, conducted in the Middle East, reported that *BRCA1* PSVs were significantly associated with positive family history and emphasized on the importance of genetic counseling, guidelines for risk assessment, early detection, and putting cost-effective screening programs in action [31].

This study has several strengths, and it opens the way for future studies investigating clinical endpoints among ovarian cancer patients in the Arabian Gulf region; one point of strength is the recruitment from multiple study sites across multiple countries in the Gulf region. This study also highlighted the importance of tissue archiving in a bio-bank / bio-repository and represents an encouraging example for oncology centers to start bio-banking. One potential limitation of our study is that, in absolute terms, the sample is relatively small, although this study recruited a substantial and sufficient sample size to conduct all planned analyses. Another limitation was that only 84% (88 out of 105) of patients had genetic testing done; this was attributed to insufficient sample, low sample quality, lack of tissue availability, or failed testing. Also, the germline testing was limited to those with somatic PSVs, so the prevalence of germline PSVs could not be computed. More patients will need to be recruited for future studies investigating longitudinal endpoints, such as survival. In addition to that, and although all efforts possible were made in order to ensure that accurate and complete data is collected for each patient, the extent to which it is practical to do so (for example, the completeness of electronic patient medical records) is not known a priori. Another possible limitation in our study is that the collected information regarding family history was obtained through a personal interview and was not confirmed by the medical records of family members in question or a formal cancer registry database. It is also worth mentioning that the Gulf region is characterized by a mixed ethnic population with Arabic predominance [32]. Data regarding parity were not collected as well. Secondary malignancies were excluded, as the primary objective of the study was to reflect the frequency of *BRCA1/BRCA2* PSVs in primary ovarian cancer patients. However, excluding breast cancer cases might have introduced bias to our findings. Finally, there is always a possibility among observational studies such as this one that unaccounted variables may result in residual bias in the planned analyses, though caution was taken when interpreting the final results.

## Conclusion

Our study showed that somatic *BRCA1/BRCA2* PSVs prevalence in Gulf Countries is notably higher than the reported global figures (2-8%). These results warrant more detailed future studies with a larger number of patients and further evaluation of germline and somatic *BRCA1/BRCA2* PSVs to better understand their impact on ovarian cancer patient outcomes in Gulf countries.

## Supplementary Information

**Additional file 1: Table 1.** Family history of Cancer.

## Data Availability

The datasets used and/or analysed during the current study available from the corresponding author on reasonable request.

## Abbreviations

DNA: Deoxyribonucleic Acid
FFPE: Formalin-fixed paraffin-embedded
FIGO: The International Federation of Gynecology and Obstetrics
H&E: Hematoxylin and Eosin
ICF: Informed consent form
KCCC: Kuwait Cancer Control Center
kg: Kilogram
NGS: Next-generation sequencing
m: Meter
OC: Ovarian cancer
PREDICT: Prevalence of BRCA1 and BRCA2 Pathogenic Sequence Variants in Ovarian Cancer Patients in The Gulf Region
PSVs: Pathogenic sequence variants
SD: Standard deviation
TCGA: The Cancer Genome Atlas
