# Supplementary material for: Prevalence of BRCA1 and BRCA2 pathogenic sequence variants in ovarian cancer patients in the Gulf region: the PREDICT study
Source: BMC Cancer. 2021 Dec 20;21:1350. doi: 10.1186/s12885-021-09094-8 (PMC8690897; doi:10.1186/s12885-021-09094-8)
Supplement: Supplementary file 1 — Additional file 1: Table 1. Family history of Cancer. [file 12885_2021_9094_MOESM1_ESM.pdf]

Additional Table 1: Family history of Cancer

Family Member	Breast Cancer		Ovarian Cancer		Other Cancer	
	N	%	N	%	N	%
**Mother**	3	2.9	1 (Peritoneal)	1.0	2	1.9
					Endometrial Cancer	1.0
					Gastric Cancer	1.0
Age/Unknown	76/Unk/Unk		84		Unk/Unkl	
**Mother's mother**	1	1.0				
Age/Unknown	60					
**Mother's sister**	3	2.9			1	1.0
					Uterine Cancer	
Age/Unknown	50/Unk/Unk				Unk	
**Father**	1					
Age/Unknown	60					
**Sister**	2	1.9	1 (Peritoneal)	1.0	1 Lung Cancer	1.0
Age/Unknown	54/Unk		Unk		Unk	
**Brother**					1 Colon Cancer	1.0
Age/Unknown					Unk	
